# Thromboelastography-based anticoagulation management during extracorporeal membrane oxygenation: a safety and feasibility pilot study

**DOI:** 10.1186/s13613-017-0352-8

**Published:** 2018-01-16

**Authors:** Mauro Panigada, Giacomo E. Iapichino, Matteo Brioni, Giovanna Panarello, Alessandro Protti, Giacomo Grasselli, Giovanna Occhipinti, Cristina Novembrino, Dario Consonni, Antonio Arcadipane, Luciano Gattinoni, Antonio Pesenti

**Affiliations:** 1 Department of Anesthesiology, Intensive Care and Emergency, Fondazione IRCCS Ca’ Granda - Ospedale Maggiore Policlinico, Milan, Italy; 2 Department of Pathophysiology and Transplantation, Università degli Studi di Milano, Milan, Italy; 3 Department of Anesthesiology and Intensive Care, ISMETT IRCCS (Istituto Mediterraneo per i Trapianti e Terapie ad Alta Specializzazione) - UPMC, Palermo, Italy; 4 Central Chemical, Clinical and Microbiology Analysis Laboratory Department of Services and Preventive Medicine, Fondazione IRCCS Ca’ Granda - Ospedale Maggiore Policlinico, Milan, Italy; 5 Epidemiology Unit, Fondazione IRCCS Ca’ Granda - Ospedale Maggiore Policlinico, Milan, Italy; 6 University of Göttingen, Göttingen, Germany

**Keywords:** Extracorporeal membrane oxygenation, Thromboelastography, Anticoagulation, Heparin, Hemorrhage, Thrombosis

## Abstract

**Background:**

There is no consensus on the management of anticoagulation during extracorporeal membrane oxygenation (ECMO). ECMO is currently burdened by a high rate of hemostatic complications, possibly associated with inadequate monitoring of heparin anticoagulation. This study aims to assess the safety and feasibility of an anticoagulation protocol for patients undergoing ECMO based on thromboelastography (TEG) as opposed to an activated partial thromboplastin time (aPTT)-based protocol.

**Methods:**

We performed a multicenter, randomized, controlled trial in two academic tertiary care centers. Adult patients with acute respiratory failure treated with veno-venous ECMO were randomized to manage heparin anticoagulation using a TEG-based protocol (target 16–24 min of the R parameter, TEG group) or a standard of care aPTT-based protocol (target 1.5–2 of aPTT ratio, aPTT group). Primary outcomes were safety and feasibility of the study protocol.

**Results:**

Forty-two patients were enrolled: 21 were randomized to the TEG group and 21 to the aPTT group. Duration of ECMO was similar in the two groups (9 (7–16) days in the TEG group and 11 (4–17) days in the aPTT group, *p* = 0.74). Heparin dosing was lower in the TEG group compared to the aPTT group (11.7 (9.5–15.3) IU/kg/h vs. 15.7 (10.9–21.3) IU/kg/h, respectively, *p* = 0.03). Safety parameters, assessed as number of hemorrhagic or thrombotic events and transfusions given, were not different between the two study groups. As for the feasibility, the TEG-based protocol triggered heparin infusion rate adjustments more frequently (*p* < 0.01) and results were less frequently in the target range compared to the aPTT-based protocol (*p* < 0.001). Number of prescribed TEG or aPTT controls (according to study groups) and protocol violations were not different between the study groups.

**Conclusions:**

TEG seems to be safely used to guide anticoagulation management during ECMO. Its use was associated with the administration of lower heparin doses compared to a standard of care aPTT-based protocol.

*Trial registration* ClinicalTrials.gov, October 22,2014. Identifier: NCT02271126.

**Electronic supplementary material:**

The online version of this article (10.1186/s13613-017-0352-8) contains supplementary material, which is available to authorized users.

## Background

During extracorporeal membrane oxygenation (ECMO), systemic anticoagulation is routinely used to avoid thrombin generation due to the contact of blood with non-biological surfaces of the extracorporeal circuit. Despite this, some degree of coagulopathy can be detected almost immediately after the start of the extracorporeal circulation [1, 2]. This may results in both bleeding and thrombotic events, which are the most frequent adverse effects during ECMO jeopardizing the final outcome [3, 4]: whether hemostasis-related complications could be attributed to: (1) the underlying patient conditions (such as sepsis or inflammation); (2) the effect of anticoagulant drugs; or (3) the suboptimal anticoagulation monitoring, is still matter of debate.

So far, we do not have many answers regarding the first two issues. Interactions between coagulation, inflammation and immune pathways are increasingly recognized but still represent an open field of research [5, 6] and unfractionated heparin remains the anticoagulant drug of choice in most ECMO centers [7, 8]. Anticoagulation monitoring methods are also far from being ideal [9]. Activated partial thromboplastin time (aPTT) is universally recognized as the standard assay for monitoring heparin therapy [10, 11] and it is considered a good choice in the adult population treated with ECMO [7]. However, aPTT is performed on plasma samples and may not provide a comprehensive picture of the hemostatic profile in the absence of the cellular components of the blood. In contrast, thromboelastography (TEG) is a whole blood viscoelastic point-of-care test, which provides information regarding the entire coagulation cascade (including platelet function, platelet–fibrin interactions with red blood cells and fibrinolysis). At present, TEG has been recommended only to manage coagulation abnormalities during ECMO [12]. Nevertheless, the TEG reaction time (the time needed to change the physical nature of blood from liquid to gel) could act as a surrogate for thrombin generation and drive heparin infusion [13–15]. In a retrospective study on 32 consecutive patients treated with ECMO for severe respiratory failure, we frequently observed a marked heparin effect on the TEG tracing despite an aPTT ratio and activated clotting time (ACT) within the therapeutic anticoagulation range [16]. Aware of these findings, since excessive anticoagulation remains a reasonable risk factor for bleeding during ECMO [17, 18], we raised the question as to whether using a TEG-driven strategy to titrate heparin during ECMO could represent a safe and feasible alternative to the “conventional” approach based on aPTT monitoring.

We designed the present pilot study to compare two protocols for managing heparin therapy during veno-venous ECMO: one based on thromboelastography and the other based on aPTT ratio, in a population of patient with severe respiratory failure.

## Methods

### Study design

This was a multicenter; prospective, randomized, pilot trial performed at two Italian referral ECMO hospitals (Fondazione IRCCS Ca’ Granda - Ospedale Maggiore Policlinico, Milan; ISMETT Istituto Mediterraneo per i Trapianti e Terapie ad Alta Specializzazione, Palermo). The study was registered at ClinicalTrials.gov with the identifier: NCT02271126. Ethics Committee at both participating centers approved the study protocol and informed consent was obtained according to Italian regulations.

### Eligible subjects

Patients requiring ECMO for acute respiratory distress syndrome (ARDS) or as a bridge to lung transplant were screened for inclusion. Exclusion criteria were veno-arterial ECMO, age < 18 years, heparin-induced thrombocytopenia (HIT) or platelet count < 30,000/mm^3^ and acute respiratory failure after lung transplant. The study ended at ECMO disconnection, lung transplant (in the case of bridge to lung transplant), death of the patient or onset of HIT.

### ECMO system

Extracorporeal membrane oxygenation was provided as femoro-femoral, femoro-jugular or jugular–femoral veno-venous bypass. Spring wire-reinforced cannulas for both drainage (21–23 Fr, HLS cannulae; MAQUET Cardiopulmonary AG) and reinfusion (21 Fr, Bio-Medicus Venous cannulae, Medtronic Inc.) were inserted percutaneously. QUADROX PLS Oxygenator and ROTAFLOW Centrifugal Pump or HLS Set Advanced 5.0—Cardiohelp system (MAQUET Cardiopulmonary AG, Hirrlingen, Germany)—were used. All circuit components, including oxygenators, centrifugal pumps and tubings, were BIOLINE coated. Membrane lungs were ventilated with an oxygen–air blender and maintained at 37 °C with a heat exchanger.

### Randomization

Patients were randomized in blocks to the aPTT or the TEG-based protocol. Randomization was performed centrally, with the use of a computer-generated and blinded assignment sequence. Randomization was stratified according to the participating ICU and to the reason for ECMO connection (ARDS or bridge to lung transplant).

### Study protocol

Before cannulation, blood samples were drawn for baseline blood chemistry and coagulation parameters. Simultaneously a fresh blood sample was also collected to measure the baseline thromboelastography (TEG^®^, Haemonetics, Braintree, MA, USA) profile, using both the kaolin reagent to start the coagulation cascade (TEG-K) and the kaolin + heparinase reagent to exclude the heparin effect (TEG-KH).

**Fig. 1:**
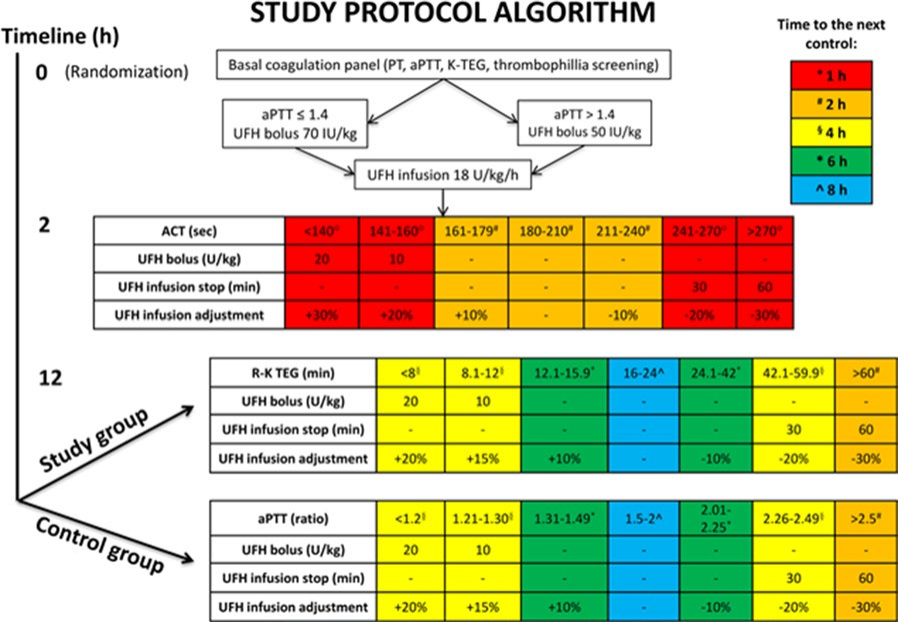
During the first 12 h after ECMO cannulation, the algorithm was identical in the two study groups. After the first 12 h according to randomization, the anticoagulation management was modified according to the assigned study group: In the TEG group (intervention group), heparin infusion was titrated to reach a target TEG-K reaction time (R-K) of 16–24 min (normal values: 4–8 min); in the aPTT group (standard of care–control group), heparin infusion was titrated to reach a target aPTT ratio value of 1.5–2. Under-target levels of anticoagulation were corrected with either heparin bolus plus increase in infusion or with increase in infusion only; similarly, over-target levels of anticoagulation were corrected with either heparin infusion stop for 30 or 60 min and restart with a reduced dose or with decrease in infusion only. Time to the next control varied according to the degree of derangement from target values of anticoagulation. In case of surgery, minimal levels of anticoagulation (i.e., R-K of 8–12 min and aPTT ratio of 1.2–1.3, in the study and control group, respectively) were tolerated during the first 24 h after the operation. In case of bleeding, heparin infusion was reduced to the lower value of the target range or interrupted based on the severity of bleeding

In both study groups, all patients initially received a bolus of unfractionated heparin (70 UI/kg if baseline aPTT ratio was < 1.4 or 50 UI/kg if it was ≥ 1.4) followed by a continuous heparin infusion (starting at 18 UI/Kg/h) to reach a target-activated clotting time (ACT, Hemocron Jr. ^®^ Signature+) between 180 and 210 s. ACT can be performed on a portable instrument; thus, it was used during the first 12 h in both groups to uniform the heparin-guided management in case of transport/centralization of a patient. After the first 12 h according to randomization, the anticoagulation management was modified according to the assigned study group: (a) In the TEG group (intervention group), heparin infusion was titrated to reach a target TEG-K reaction time (R-K) of 16–24 min (normal values: 4–8 min) and (b) in the aPTT group (standard of care–control group), heparin infusion was titrated to reach a target aPTT ratio value of 1.5–2. In both groups, heparin infusion rate was then adjusted according to a predefined algorithm (Fig. 1) by using only TEG or aPTT, respectively. In case of a “flat-line” TEG (i.e., a TEG tracing unable to generate a R-K parameter without heparinase within 90 min), R-K was censored at 90 min. To study the correlation between R-K and aPTT, in the TEG group one single aPTT value was obtained in the morning; similarly, one single determination of R-K was obtained every morning in the aPTT group. In case of surgery, minimal levels of anticoagulation (i.e., R-K of 8–12 min and aPTT ratio of 1.2–1.3, in the study and control group, respectively) were tolerated during the first 24 h after the operation. In case of bleeding heparin infusion was reduced to the lower value of the target range or interrupted based on the severity of bleeding. Heparin dose was never increased in the TEG group if the corresponding aPTT was 2.5 or higher.

Every violation of the study protocol was recorded. Violations were classified as:

- minor violations: inadvertent violations and significant (> 4 h) delays in TEG or aPTT analysis
- major violations: intentional violations by the medical staff for clinical reasons.

Hemoglobin concentration, platelet count, prothrombin time (PT) (patient-to-normal) ratio, fibrinogen and D-dimer level were determined every 8 h in all patients. Free hemoglobin level, haptoglobin, antithrombin and anti-factor Xa activity (anti-Xa) were measured once daily in all patients. Packed red blood cells, fresh frozen plasma and platelets were transfused to maintain hemoglobin concentration > 10 g/dL, PT ratio < 1.5 and platelet count > 45,000/mm^3^. Fibrinogen and antithrombin were supplemented to maintain fibrinogen levels > 150 mg/dL and antithrombin activity > 70%. Transfused blood products were registered every day as well as the total amount of heparin infused to each patient (including boluses and holding of the infusion).

ECMO circuit (including the artificial lung) function was regularly assessed with a dedicated score (circuit change score, Additional file 1: Table E1). Circuit change was performed when the circuit change score was > 5 *and* there was evidence of bleeding from any site (Additional file 1: Figure E1).

### Outcomes

Primary outcomes of the study were:

1. Safety of the study protocol: number of hemorrhagic or thrombotic events and blood product transfusions.

Bleeding was regularly assessed using a checklist of potential bleeding sites. We also standardized severity of bleeding using five categories adjusted from the Bleeding Academic Research Consortium score [19]:

- Type 0: No bleeding;
- Type 1: Any overt bleeding that requires heparin infusion rate reduction *or* packed red blood cells transfusion (provided hemoglobin drop was related to bleeding);
- Type 2: Any overt bleeding that requires heparin infusion rate reduction *and* packed red blood cells transfusion (provided hemoglobin drop was related to bleeding);
- Type 3: Any life-threatening bleeding that required packed red blood cells transfusion and surgical intervention for control of bleeding or ECMO discontinuation;
- Type 4: Any fatal bleeding.

Any objective sign of patient’s thrombosis was reported everyday at daily visit. The extracorporeal circuit and the ECMO oxygenator were inspected three times a day in order to detect visible clots. Within 24 h after ECMO removal, a Doppler ultrasonography of the cannulated vessels and of the vena cava was performed to exclude thrombosis due to vascular cannulation.

(2) Feasibility of the study protocol: number of heparin dose adjustments, number of prescribed analyses, number of R-K or aPTT ratio (according to groups) results within the target range and number of study protocol violations.

Secondary outcomes were number of ECMO circuit failures/changes, over-anticoagulation (expressed as the proportion of flat-line TEG) and correlation between heparin dose and aPTT or R-K in the two groups.

Finally, we performed a cost analysis, to compare the expenses for monitoring tests execution and heparin infusion between the study groups.

### Statistical analysis

Given the nature of this study (pilot), a sample size of 12 patients per group is considered sufficient [20]. To increase power, we foresaw to enroll at least 20 patients per group. Continuous variables are reported as median and 25th and 75th percentile. Categorical variables are presented as absolute and relative frequencies. Wilcoxon rank-sum tests and Fisher’s exact test were used to analyze continuous and categorical variables, respectively. Correlations between heparin dose and monitoring methods were expressed using the Spearman correlation coefficient. To compare blood and coagulation parameters in the two groups during the study period, we used random intercept models because the outcomes were repeated within subjects [21]. We used a multinomial logistic regression model to calculate the association between the proportion of aPTT and R-K within target range in the two study groups. All tests were two-sided. Stata 13.1 was used for analyses (StataCorp, College Station, TX, USA).

## Results

**Fig. 2:**
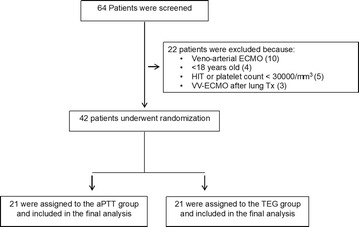
Flow diagram. We assessed 64 patients for eligibility. Of these, patients 22 were excluded. We enrolled and randomly assigned the remaining 42 to the aPTT or TEG arm. Thirty-one patients were enrolled in the Fondazione Ca’ Granda (Milan, Italy) center and 11 patients in the ISMETT (Palermo, Italy) center

Between September 2, 2014, and November 30, 2016, we screened 64 patients; of these, 42 were enrolled (21 in the TEG group and 21 in the aPTT group). (In Fig. 2 reasons for exclusion of the remaining patients are depicted.) Baseline clinical characteristics and coagulation parameters of the patients are summarized in Table 1. There appeared to be a degree of imbalance only in the proportion of women enrolled in the two groups. There was no difference in baseline coagulation parameters; only one patient for each group presented with DIC at study entry. C-reactive protein was not different in the two groups at study entry: 16.2 (8.2–24.1) mg/dL in the TEG group and 14.5 (6.7–25.2) mg/dL in the aPTT group, *p* = 0.63.

**Table 1:** Characteristics of the patients and baseline coagulation parameters

Characteristics of the patients	TEG group (*N* = 21)	aPTT group (*N* = 21)	*P* value*
Age (years)	43 (36–53)	48 (40–58)	0.24
Female sex, *N* (%)	13 (62)	5 (24)	0.03
BMI (kg/m^2^)	21.8 (20.8–26.1)	27.7 (21.2–29.4)	0.11
ECMO duration (days)	9 (7–16)	11 (4–17)	0.74
Cause of respiratory failure			0.73
ARDS, *N* (%)	14 (66.7)	16 (76.2)	
Bridge to lung transplant, *N* (%)	6 (28.6)	5 (23.8)	
Status asthmaticus, *N* (%)	1 (4.8)	0	
Renal replacement therapy, *N* (%)	4 (19)	7 (33)	0.48
SOFA	7 (5–10)	9 (6–11)	0.54
ICU mortality, *N* (%)	4 (19)	6 (29)	0.72
Hospital mortality, *N* (%)	4 (19)	6 (29)	0.72
Baseline coagulation parameters			
Platelet count (*10^9^/L)	185 (81–205)	154 (109–268)	0.77
Hemoglobin (g/L)	10.6 (9–11.1)	11.2 (9.8–12.3)	0.09
Creatinine (mg/dL)	0.9 (0.4–1.0)	1.1 (08.1.62)	0.20
Fibrinogen (mg/dL)	520 (350–626)	476 (357–573)	0.77
PT (ratio)	1.3 (1.2–1.4)	1.3 (1.1–1.5)	0.87
DIC°, *N* (%)	1 (4.8)	1 (4.8)	0.76
DIC score°	1 (1–3)	3 (2–3)	0.33

Data are reported as median (25th–75th percentile) or as absolute and relative frequencies as appropriate

*BMI* body mass index, *ARDS* acute respiratory distress syndrome, *SOFA* sepsis-related organ failure assessment, *PT* prothrombin time, *DIC* disseminated intravascular coagulation, *ICU* intensive care unit

*From Wilcoxon rank-sum (continuous variables) or Fisher’s exact (categorical variables) test

°ISTH criteria (Taylor, FB et al. Thromb Haemost. 2001). DIC patients: #4 (aPTT group) and #30 (TEG group)

Table 2 shows blood and coagulation parameters during the study period. Heparin infusion rate, aPTT ratio, R-K and anti-Xa were lower in the TEG group compared to the aPTT group.

**Table 2:** Blood parameters, coagulation parameters and anticoagulation in the two groups during the study period

Parameters	TEG group (*N* = 21)	aPTT group (*N* = 21)	*P* value*
Hemoglobin (g/dL)	10.0 (9.5–10.6)	10.2 (9.7–10.8)	0.25
Hematocrit (%)	30.5 (28.5–31.9)	30.8 (29.0–32.4)	0.47
Platelet count (*10^9^/L)	118.0 (72–202)	122 (88–172)	0.78
Creatinine (mg/dL)	0.6 (0.4–1.1)	1.0 (0.8–1.3)	0.14
Heparin^§^ (IU/kg/h)	11.7 (9.5–15.3)	15.7 (10.9–21.3)	0.03
aPTT (ratio)	1.2 (1.1–1.6)	1.6 (1.4–1.8)	< 0.001
R-K° (min)	18.8 (12.9–27.4)	61.9 (32.7–90.0)	< 0.001
Flat-line TEG, *N* (%)	34 (4.7)	52 (37.7)	< 0.001
R-KH (min)	6.2 (5.2–7.6)	8.1 (6.8–9.9)	0.50
Anti-Xa (IU/mL)	0.1 (0.1–0.2)	0.3 (0.2–0.4)	< 0.001
PT (ratio)	1.1 (1.1–1.3)	1.2 (1.1–1.3)	0.69
Antithrombin (%)	90.0 (71.0–103.0)	77.0 (64.0–91.0)	0.15
Antithrombin (IU/die)	1000 (0–2000)	2000 (2000–2000)	0.17
Fibrinogen (mg/dL)	391.5 (275.0–524.5)	466.0 (327.0–611.0)	0.96
Free hemoglobin (mg/dL)	8.5 (6.7–10.4)	9.1 (7.0–12.7)	0.50
CRP (mg/dL)	12.5 (7.6–23.0)	15.3 (7.8–23.2)	0.52

Data are reported as median (25th–75th percentile) or as absolute and relative frequencies as appropriate

Heparin and antithrombin were administered i.v. as continuous infusion

*N* number of measurements, *PT* prothrombin time, *aPTT* activated partial thromboplastin time, *CRP* C-reactive protein

*From random intercept linear regression models

^§^The amount of heparin infused to each patient was calculated as the total administered heparin per kg of body weight (including boluses) divided by the total hours of heparin infusion. *P* value from Wilcoxon rank-sum test

°R-K was arbitrarily considered equal to 90 if unable to generate a value within 90 min (flat-line)

Duration of ECMO was comparable between the two groups: 9 (7–16) days in the TEG group and 11 (4–17) days in the aPTT group, *p* = 0.74.

### Safety outcomes

Hemorrhagic and thrombotic complications are reported in Table 3. Although not significantly, patients in the aPTT group tended to bleed more compared to the TEG group (15 vs. 10, respectively, *p* = 0.21) especially in surgical sites (including bleeding from tracheostomy) (*p* = 0.02). The most frequent hemorrhagic complication was mucosal bleeding in both groups. None of the patients experienced intracranial bleeding. Severity of bleeding episodes was not different between the two groups (*p* = 0.62). Most of the patients were classified with a “bleed type = 0” (9 vs. 11 patients in the aPTT and TEG group, respectively), and 4 patients were classified with a bleed type ≥ 3 in the aPTT group and 1 in the TEG group.

**Table 3:** Hemorrhagic and thrombotic complications

Complications	TEG group (*N* = 21)	aPTT group (*N* = 21)	*P* value*
Patients with any bleeding, *N* (%)	10 (47.6)	15 (71.4)	0.21
Site of bleeding^#^, *N* (%)			
CNS	0 (0)	0 (0)	–
Gastrointestinal tract	3 (14.3)	1 (4.8)	0.61
Vascular catheters insertion site	4 (19.0)	7 (33.3)	0.48
ECMO cannula insertion site	5 (23.8)	5 (23.8)	1.0
Mucosal bleeding (oral, nasal, airways)	6 (28.6)	10 (47.6)	0.34
Surgical site (including tracheostomy)	0 (0)	6 (28.6)	0.02
Urinary tract	2 (9.5)	5 (23.8)	0.41
Severity of bleeding (bleed type)			0.46
Type 0, *N* (%)	11 (52.38)	9 (42.86)	
Type 1, *N* (%)	1 (4.76)	3 (14.29)	
Type 2, *N* (%)	8 (38.10)	5 (23.81)	
Type 3, *N* (%)	1 (4.76)	3 (14.29)	
Type 4, *N* (%)	0 (0.00)	1 (4.76)	
Patients with any thrombosis, *N* (%)	4 (19.0)	4 (19.0)	1.0
Site of thrombosis, *N* (%)			
Deep venous thrombosis (ECMO cannula-related)	2 (9.5)	3 (14.3)	1.0
Vascular catheter-related thrombosis	1 (4.8)	1 (4.8)	1.0
ECMO cannula occlusion	1 (4.8)	0	1.0
RBC transfused (ml/day/patient)	198 (37–330)	203 (155–247)	0.74
FFP transfused (ml/day/patient)	0 (0–79)	0 (0–0)	0.54
Platelet transfused (ml/day/patient)	0 (0–61)	0 (0–0)	0.28

Data are reported as median (25th–75th percentile) or as absolute and relative frequencies as appropriate

*CNS* central nervous system, *ECMO* extracorporeal membrane oxygenation, *RBC* red blood cells, *FFP* fresh frozen plasma

^#^A patient could have more than one site of bleeding

^§^Categories of bleeding according to Mehran et al. [18]

*From Wilcoxon rank-sum (continuous variables) or Fisher’s exact (categorical variables) test

Four patients in both groups experienced thrombotic complications, without differences in specific sites of thrombosis. The most frequent thrombotic complication was deep venous thrombosis related to the presence of the ECMO cannula in both groups. D-dimer was not different in the two groups during the study time (*p* = 0.95) (Additional file 1: Table E2).

Transfusions of blood products were not different in the study groups.

### Feasibility outcomes

**Fig. 3:**
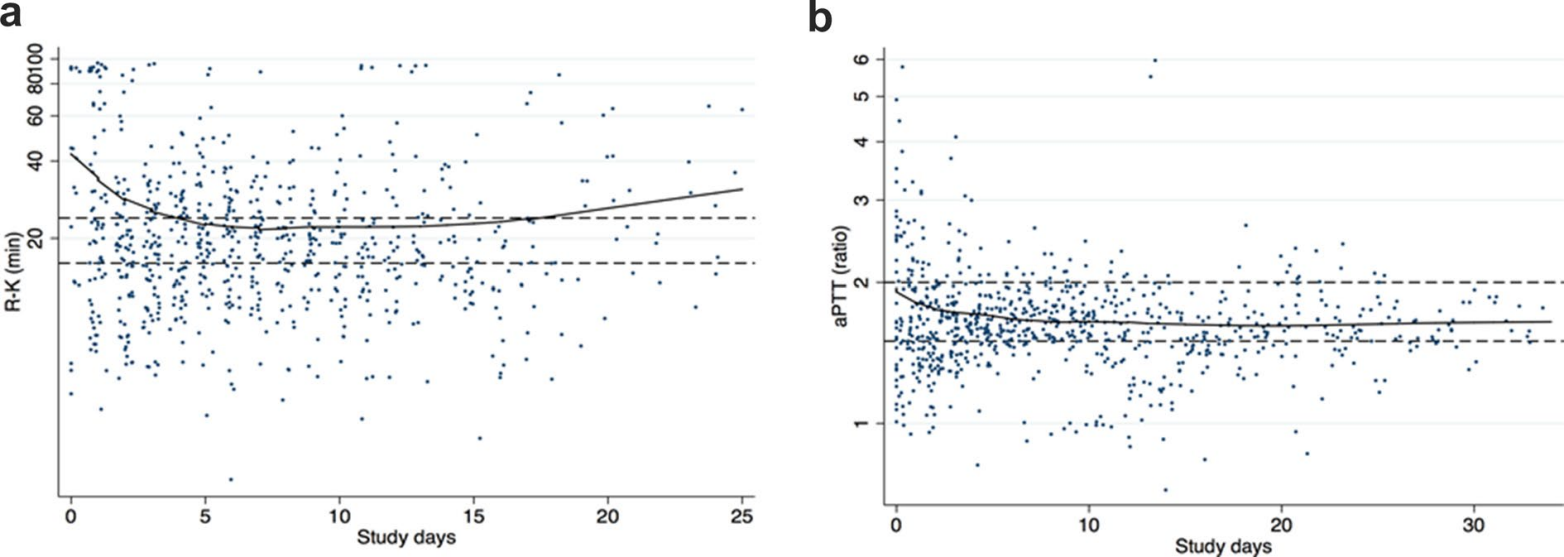
aPTT ratio and R-K values during study days in patients randomized to the aPTT group (panel A) and to the TEG group (panel B). Lines are lowess smoothing. Reference lines: 1.5–2 for the aPTT ratio and 16–24 min for the R time. 56.5% of the analysis was in range in the aPTT group compared to 29.8% in the TEG group (*p* < 0.001). *aPTT* activated partial thromboplastin time ratio, *R-K* TEG reaction time, *TEG* thromboelastography

TEG results triggered a heparin infusion rate change more frequently than aPTT results (0.58 (0.53–0.71) vs. 0.40 (0.30–0.53) heparin rate changes per analysis, respectively, *p* = 0.01). The number of TEG or aPTT controls (according to study groups) to each patient per day was not different in the two groups [3.2 (2.8–3.4) TEG analyses in the TEG group compared to 3 (2.9–3.3) aPTT analyses in the aPTT group (*p* = 0.88)]. Analyses were in the target range (i.e., R-K 16–24 min for the TEG group and aPTT ratio 1.5–2 for the aPTT group) more frequently in the aPTT group compared to the TEG group, 56.5 and 29.8% of the times, respectively (*p* < 0.001). In the latter group, R-K values were more frequently close or above the upper limit of the target values (Fig. 3).

Major violations to the protocols algorithms were [0 (0–0.1) per patient per day in each group and 0.1 (0–0.2) and 0 (0–0.2) minor violations in the TEG and aPTT groups, respectively (*p* = 0.52)].

### Secondary outcomes

The ECMO circuit was never exchanged in the vast majority of patients in both study groups (0 (0–1) and 0 (0–0) circuit per patient in the TEG and aPTT group, respectively, *p* = 0.82). There was no difference in the circuit change score between the two study groups either (4 (1–5), max 13, in the TEG group and 3 (1–5), max 8, in the aPTT group, *p* = 0.35). The proportion of flat-line TEG results was lower in the TEG group compared to the aPTT group (4.7 vs. 37.7%, *p* < 0.001). In both study groups, heparin dose was neither correlated with R-K (Spearman’s rho = 0.16, *p* = 0.29 from random intercept model) nor with aPTT (Spearman’s rho = 0.23, *p* = 0.46 from random intercept model). R-K was significantly and moderately correlated with anti-Xa only when anti-Xa was below the anticoagulant range (< 0.3 IU/mL) (Spearman’s rho = 0.53, *p* = 0.01 from random intercept model). The median anti-Xa of flat-line TEG tracings was 0.36 (0.26–0.41) IU/mL.

Following the TEG algorithm was more expensive than following the aPTT algorithm [26.28 (23.86–28.36) € vs. 8.70 (8.53–9.49) € per patient per day, respectively, *p* < 0.001]. The use of the TEG algorithm resulted in a lower expense for heparin (3.97 (2.78–5.20) € vs. 6.79 (5.18–7.98) € per patient per day, respectively, *p* < 0.001).

## Discussion

In our pilot study, an anticoagulation managing strategy based on a thromboelastography-derived parameter, *R* time, seems to be as safe as the “conventional” strategy based on aPTT ratio monitoring. Moreover, although there was no (statistically significant) difference in hemorrhagic complications and blood product transfusions between the two study groups, we observed a tendency for less bleeding in the TEG group, with no difference in thrombosis. We also observed a lower incidence of bleeding from surgical sites in the TEG group.

As for the feasibility, the number of protocol violations was small in both groups, indicating that the algorithm was easy to follow. Interestingly, the number of analyses in the target range was lower in the TEG group, resulting in a higher number of heparin dosing adjustments. This may be explained by the sensitivity of TEG (even in the early phases of clot formation) to low heparin dosing [11] and to fibrinogen and platelet levels [22].

As expected from previous research [16], our TEG-based protocol resulted in the prescription of a much lower amount of heparin, even inferior to usual prophylactic doses. This was confirmed by significantly lower values in the TEG group of anti-Xa activity, which is considered the gold standard assay to measure heparin effect; importantly, in our study this test was performed without adding antithrombin to the plasma sample. It must be noted, however, that anti-Xa values were at the lower end of the recommended level for proper anticoagulation [12, 23] even in the aPTT group. Nevertheless, the number of thrombotic episodes was small and not different between the study groups.

Since this was the first time a TEG-based protocol was used to adjust heparin infusion, we planned a safety rule of not increasing heparin dose in the TEG group if the corresponding aPTT was 2.5 or higher. Yet, this never happened.

One of the major concerns of using a low level of anticoagulation during ECMO is the development of consumptive coagulopathy [1, 24]; this was not the case since D-dimer levels were comparable in the two groups. Importantly, we tried to standardize the timing of circuit exchange by means of daily calculation of a “circuit change score” based on coagulation alterations, heparin requirements, visual evaluation of clotting in the system and functional ECMO parameters. This score together with the evidence of clinically relevant coagulation alterations (i.e., bleeding), prompted the decisions to exchange the circuit. This may have prevented the development of a severe form of coagulopathy. However, score values and the number of exchanged ECMO system were similar in the two groups.

Since excessive anticoagulation increases the risk of bleeding during ECMO [17] and advances in technology (i.e., circuit coating with biocompatible surfaces) are supposed to reduce the incidence of circuit thrombosis, several reports of minimal anticoagulation during ECMO have been published. In a retrospective study by Agerstrand et al., incidence of bleeding was reduced and circuit or oxygenator thrombosis was rare, using a low target aPTT [25]. Yeo et al. described a lower incidence of bleeding and blood products transfusions in patients anticoagulated according to a lower than conventional ACT target [26]. Recently, Krueger et al. reported that a prophylactic regimen with only subcutaneous enoxaparin was feasible and safe in 61 patients on veno-venous ECMO [27]. Anyhow, due to the high variability in patients’ response to heparin [28], we believe in the added value of monitoring anticoagulation also when low levels of heparin are used. In our study, we confirmed that R-K is sensitive to low doses of heparin, but it loses accuracy at recommended anticoagulant ranges (i.e., anti-Xa 0.3–0.7 IU/mL) [23, 29]. Interestingly, the frequency of flat-line TEG tracings was lower in this study compared to our previous experiences [16]. This may be explained by a strict and prospective adherence to the protocol for heparin adjustment, which was not the case in our previous retrospective study.

Since the anticoagulant effect of heparin is mediated mainly by antithrombin [10], we supplemented antithrombin to achieve an activity level above 70%. We observed a tendency for lower antithrombin levels in the aPTT group, probably explained by increased consumption mediated by higher administered heparin dose.

Our study revealed that the use of TEG resulted in a higher costs compared to the aPTT group. Performing a TEG in fact is more expensive than requesting an aPTT from the laboratory, at least in the two facilities where the study was conducted. Notwithstanding, this was balanced in part by the lower expenses for the anticoagulant.

Our study has several limitations. First, it is a pilot study on a relatively small number of patients. Second, the target ranges for anticoagulation applied in both groups were arbitrarily chosen. Indeed, anticoagulation targets are different between ECMO centers [8]. We used an aPTT range between 1.5 and 2 times control, which is considered adequate in other settings [28] [30, 31] and that was common practice in the two participating institutions; however, it has never been validated during ECMO. Likewise the target R-K time of 16–24 min (equivalent to 2–3 times the upper limit of normal values) had not been validated before. Both of them may require to be better defined. Third, the use of FFP and antithrombin, the target hemoglobin, fibrinogen and platelets is not standard across centers. Hence, the results of our study might have also been influenced by the contributions of these variables.

## Conclusions

We found that using a TEG-driven protocol seems to be feasible to manage heparin anticoagulation during VV-ECMO and does not look to be associated with an increased rate of complications. Furthermore, even if our study was not powered to detect major differences in outcomes, the TEG protocol allowed the administration of lower heparin doses without an increase in thrombotic complications and with a tendency for less bleeding compared to the aPTT protocol. A larger trial is warranted to confirm these preliminary findings.

## Additional file

**Additional file 1.** Score and flow diagram for ECMO circuit change.

## Abbreviations

ACT: activated clotting time
Anti-Xa: anti-factor Xa activity
aPTT: activated partial thromboplastin time
ARDS: acute respiratory distress syndrome
ECMO: extracorporeal membrane oxygenation
HIT: heparin-induced thrombocytopenia
TEG: thromboelastography
TEG-K: kaolin-activated thromboelastography
TEG-KH: kaolin-activated thromboelastography with heparinase
PT: prothrombin time
R: thromboelastography reaction time
R-K: kaolin-activated thromboelastography reaction time
