# Supplementary material for: Thromboelastography-based anticoagulation management during extracorporeal membrane oxygenation: a safety and feasibility pilot study
Source: Ann Intensive Care. 2018 Jan 16;8:7. doi: 10.1186/s13613-017-0352-8 (PMC5770349; doi:10.1186/s13613-017-0352-8)

**Supplementary Material**

**Thromboelastography-based anticoagulation management during extracorporeal membrane oxygenation: a safety and feasibility pilot study.**

Mauro Panigada^1^, MD; Giacomo Iapichino^2^, MD; Matteo Brioni^2^, MD; Giovanna Panarello^3^, MD; Alessandro Protti^1^, MD; Giacomo Grasselli^1^, MD; Giovanna Occhipinti^3^, MD; Cristina Novembrino^4^, PhD; Dario Consonni^5^, MD; Antonio Arcadipane^3^, MD; Luciano Gattinoni^6^, MD; Antonio Pesenti^1^, MD.

^1^ Fondazione IRCCS Ca' Granda - Ospedale Maggiore Policlinico, Milano, Italy - Department of Anesthesiology, Intensive Care and Emergency

^2^ Università degli Studi di Milano, Milano, Italy - Department of Pathophysiology and Transplantation

^3^ ISMETT IRCCS (Istituto Mediterraneo per i Trapianti e Terapie ad Alta Specializzazione) - UPMC, Palermo, Italy - Department of Anesthesiology and Intensive Care

^4^ Fondazione IRCCS Ca’ Granda - Ospedale Maggiore Policlinico, Milano, Italy - A.Bianchi Bonomi Hemophilia and Thrombosis Center, Laboratory of Clinical Chemistry and Microbiology

^5^ Fondazione IRCCS Ca' Granda - Ospedale Maggiore Policlinico, Milano, Italy – Epidemiology Unit

^6^ University of Göttingen, Göttingen, Germany

**Additional file 1**

**Table E1 - Circuit change score**

| **Platelet count*$** | ↓ 50% in 48 h =  1 point | ↓ 50% in 24 h =  2 points | ↓ 50% in 48 h and < 45000 u/mL =  3 points | ↓ 50% in 24 h and < 45000 u/mL =  4 points |
| --- | --- | --- | --- | --- |
| **Fibrinogen$** | -- | -- | ↓ 50% in 48 h and < 150 mg/dL =  1 point | ↓ 50% in 24 h and < 150 mg/dL =  2 points |
| **Artificial lung performance** | TMP 30-50 mmHg at 3 L/min = 1 point | TMP > 50 mmHg at 3 L/min = 2 points | PO_2_ post-lung < 300 mmHg (FiO2 1.0) = 1 point | PO_2_ post-lung < 200 mmHg (FiO2 1.0) = 2 points |
| **Artificial lung inspection** | -- | Clot diameter > 5 cm = 1 point | Clot diameter > 5 cm and doubling (10 cm) in < 24 h = 2 points | -- |
| **D-dimer ^$^** | < 10000 ng/mL and doubling in ≤ 24 h =  1 point | > 10000 < 20000 ng/mL = 2 points | > 10000 ng/mL and doubling in ≤ 24 h =  3 points | ≥ 20000 ng/mL and doubling in ≤ 24 h = 4 points |
| **Heparin** | + 50% daily dose = 1 point | + 100% daily dose =  2 points | -50% daily dose = 1 point | - 100% daily dose =  2 points |

Hemolysis due to ECMO circuit (Hb free > 50 mg/dl): 5 points.

* Exclude Heparin Induced Thrombocytopenia (HIT) or hemorrhage.

^$^ Rule out other possible causes of thrombocytopenia (i.e. hemorrhage, sepsis or thrombosis)

TMP = trans-membrane pressure gradient

PO_2_ = partial pressure of oxygen

**Table E2 - D-dimer categories in the two study groups**

| **Categories** | **aPTT** | **TEG** | **P value*** |
| --- | --- | --- | --- |
| 0 (< 2500 ng/ml) | 179 (24.76) | 176 (26.39) | Base |
| 1 (>2500 and < 5000 ng/ml) | 157 (21.72) | 125 (18.74) | 0.61 |
| 2 (>5000 and < 10000 ng/ml) | 196 (27.11) | 187 (28.04) | 0.95 |
| 3 (> 10000 ng/ml) | 191 (26.42) | 179 (26.84) | 0.93 |

* from multinomial logistic regression model (Wald test, p=0.95)

**Figure E1: Flow diagram for circuit change decision.**


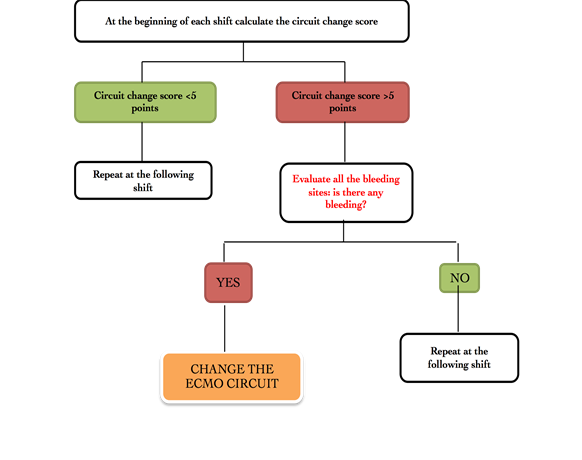

Supplement: Supplementary file 1 — Additional file 1. Score and flow diagram for ECMO circuit change. [file 13613_2017_352_MOESM1_ESM.docx]
